# Admission albumin-globulin ratio associated with delayed cerebral ischemia following aneurysmal subarachnoid hemorrhage

**DOI:** 10.3389/fneur.2024.1438728

**Published:** 2024-10-30

**Authors:** Xiumei Guo, Yu Xiong, Wen Gao, Xinyue Huang, Hanlin Zheng, Huiqiang Wu, Xutang Jiang, Qingxin Lin, Yinfeng Xiao, Qiaoling Liu, Zhigang Pan, Chunhui Chen, Weipeng Hu, Pantelis Stavrinou, Aihua Liu, Lingxing Wang, Feng Zheng

**Affiliations:** ^1^Department of Neurology, The Second Affiliated Hospital, Fujian Medical University, Quanzhou, Fujian, China; ^2^Department of Neurosurgery, The Second Affiliated Hospital, Fujian Medical University, Quanzhou, Fujian, China; ^3^Medical Center for Neurological Disorders, The Second Affiliated Hospital, Fujian Medical University, Quanzhou, Fujian, China; ^4^Department of Clinical Laboratory, the Second Affiliated Hospital of Fujian Medical University, Quanzhou, China; ^5^Department of Neurosurgery, Center for Neurosurgery, Faculty of Medicine and University Hospital, University of Cologne, Cologne, Germany; ^6^Neurosurgery, Metropolitan Hospital, Athens, Greece; ^7^Beijing Neurosurgical Institute, Beijing Tiantan Hospital, Capital Medical University, Beijing, China; ^8^Department of Neurosurgery, Ningxia Hui Autonomous Region People's Hospital, Yinchuan, China

**Keywords:** aneurysmal subarachnoid hemorrhage, delayed cerebral ischemia, serum albumin to globulin ratio, lactate dehydrogenase, phosphorous, plasma fibrinogen

## Abstract

**Background:**

Despite the widespread use in ischemic stroke, cancer, and malnutrition, the predictive ability of serum albumin to globulin ratio (A/G) among patients suffering from aneurysmal subarachnoid hemorrhage (aSAH) remains unknown. This study aimed to determine if serum A/G ratio is associated with the occurrence of delayed cerebral ischemia (DCI) after aSAH.

**Methods:**

We retrospectively viewed the medical records of aSAH patients from 08/2017 to 08/2022. Serum albumin and globulin laboratory test results were collected within 24 hours after admission. Serum A/G were dichotomized based on whether the DCI occurred. Logistic regression was used to determine the predictors of DCI. The relationship between serum A/G and the occurrence of DCI was analyzed with receiver operating characteristic(ROC) curve.

**Results:**

A total of 363 eligible patients with aSAH were included in the study, among which DCI occurred in 87 patients(23.97%). Serum A/G[OR=2.720, 95%CI (1.190-6.270), P=0.018], non-surgical[OR=0.228, 95%CI (0.065-0.621), P=0.008], lactate dehydrogenase[OR=1.004, 95%CI (1.000-1.008), P=0.029], P[OR=0.354, 95%CI (0.130-0.926), P=0.038], plasma fibrinogen[OR=1.266, 95%CI (1.019-1.583), P=0.035] were associated with the occurrence of DCI. ROC showed that serum A/G, non-surgical, LDH, P, plasma fibrinogen could predict the occurrence of DCI in aSAH patients with values 0.575, 0.560, 0.602, 0.571 and 0.539 for serum A/G, non-surgical, LDH, P, plasma fibrinogen, respectively.

**Conclusions:**

In conclusion, serum A/G levels are correlated with DCI in individuals with aSAH, and high serum A/G levels on admission may be associated with the occurrence of DCI.

## 1 Introduction

Subarachnoid hemorrhage (SAH) poses a serious risk to public health worldwide, with a mortality rate of approximately 50%, primarily attributed to ruptured intracranial aneurysms (1). Despite advances in neurocritical care, aneurysmal SAH (aSAH) is associated with a greater burden to society and a decreased quality of life (2). Delayed cerebral ischemia (DCI) is the most prevalent complication in patients with aSAH, affecting up to 30% of survivors 4–14 days after intracranial aneurysm rupture (3). Moreover, DCI is a preventable factor that leads to poor prognosis and a decreased quality of life among aSAH survivors (3). Therefore, predicting DCI in patients with aSAH is crucial (4).

Serum albumin plays essential roles in several physiological processes; it has diverse biological effects and is a predictor of nutritional status (5). Albumin protects against disorders of the central nervous system, such as ischemic stroke, Alzheimer’s disease, and SAH (6). In addition to albumin levels, inflammatory responses after SAH are associated with poor functional outcomes (7). The serum albumin to globulin ratio (A/G), determined by dividing serum albumin values by serum globulin values, is an indicator of inflammation and nutrition and is involved in predicting cancer (8), severe liver disease (9), malnutrition (10), and rheumatic disease. Serum A/G levels are associated with cognitive decline (11), and lower blood A/G ratios have been linked to poor functional outcomes and mortality in patients with ischemic stroke (12). Nonetheless, the relationship between serum A/G ratio and DCI after aSAH is uncertain. Therefore, this study aimed to determine whether blood A/G levels are associated with the development of DCI in patients with aSAH.

## 2 Materials and methods

### 2.1 Design and patients

**Figure 1:**
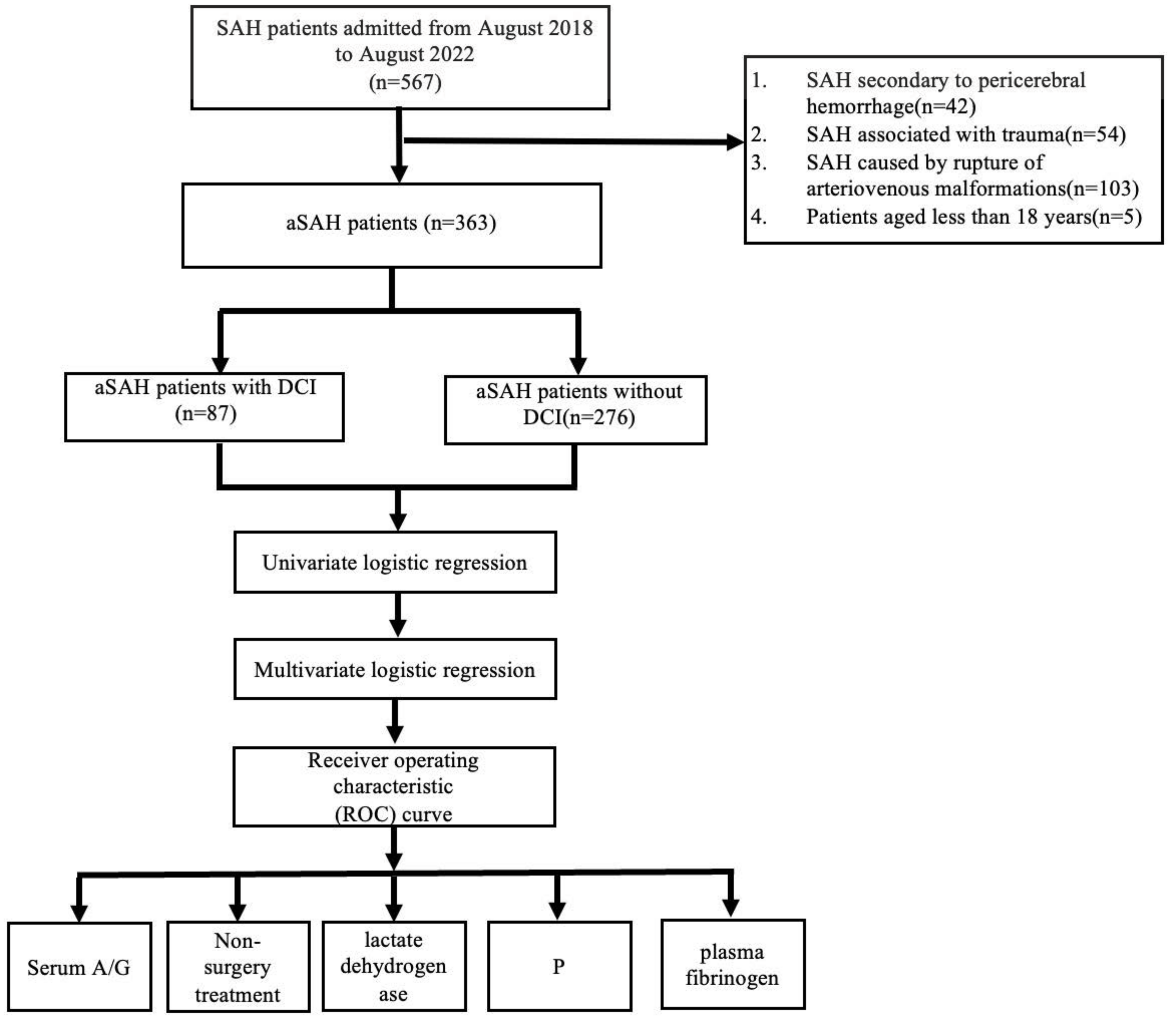
Flow chart of patient profile. DCI, delayed cerebral ischemia; SAH, subarachnoid hemorrhage; aSAH, aneurysmal subarachnoid hemorrhage; A/G, serum albumin to globulin ratio.

This single-center, observational, respective cohort study included 363 eligible patients (mean age, 57 y; women, *n* = 207 [57.0%]) with aSAH (Figure 1) among whom, 87 had DCI. These patients were admitted to the Second Affiliated Hospital of Fujian Medical University between August 2017 and August 2022 and data collected for research purposes was accessed in September 2022. This study complied with the Reporting of Enhanced Observational Studies in Epidemiology guidelines (13). We confirmed SAH upon admission using lumbar puncture and computed tomography (CT) according to these guidelines (14). Ruptured brain aneurysms causing SAH were confirmed using CT angiography (CTA) or digital subtraction angiography (DSA). Patients with SAH secondary to perimesencephalic hemorrhage, SAH associated with trauma, rupture of arteriovenous malformations, or other causes, and those aged ≤18 years were excluded from this study. The Institutional Review Board of Fujian Medical University’s Second Affiliated Hospital in China (Approval no: 623/2023) approved the protocol of the study and waived informed consent requirements because of the retrospective nature of the study.

### 2.2 Data collection and interpretation

We reviewed patient electronic medical records and obtained laboratory, clinical, and demographic information from our secure in-house database. We diagnosed DCI based on published guidelines (3), and considered focal neurological impairment (aphasia, hemiplegia, and hemi blindness) or at least a 2-point reduction in either total Glasgow Coma Scale (GCS) scores (or scores for individual components, namely, eye-opening and speech responses, and limb movement), and deemed not attributable to other clinical causes, brain imaging, and laboratory findings. Cerebral infarction was defined as a new infarct detected on CT that was not evident on images acquired immediately after admission or intervention. Other outcomes measured included pulmonary and intracranial infection, acquired epilepsy, hydrocephalus, anemia, hyperlipidemia, and cardiac insufficiency.

The baseline characteristics determined from medical records comprised age, sex, medical history (stroke, hypertension, diabetes, myocardial infarction, smoking, and alcohol consumption), systolic blood pressure (SBP) upon admission, pupillary state, aneurysm location (anterior or posterior circulation), surgical clipping, or embolization. Two neurosurgeons (ZP and XH) collected the Hunt–Hess, World Federation of Neurological Societies Scale (WFNS), and GCS clinical scores for all patients upon admission as per standard protocol. Laboratory data collected within 24 h after admission comprised routine biochemical, blood, and coagulation function indices.

### 2.3 Statistical analysis

All data were analyzed using SPSS version 28.0 (IBM Corp. Armonk, NY, USA). Descriptive characteristics are expressed as numbers and ratios (%), medians, and interquartile ranges (IQRs). Nominal variables were assessed using Fishers exact or Pearson’s chi-square tests. Continuous variables were assessed using Mann–Whitney or Kruskal–Wallis tests depending on their distribution. DCI predictors were assessed using logistic regression. Variables with a *p*-value <0.1 in the univariate logistic regression analyses were included in the multivariate logistic regression models. In the final multivariate model, predictor variables with a *p*-value <0.05 were considered to have a significant association. Additionally, to handle the missing data in this study, multiple imputation was employed in the multivariable model. Laboratory values were adjusted using covariates. The relationship between the A/G and DCI was analyzed using receiver operating characteristic (ROC) curves and the ideal cut-off value was determined using the Youden index. Values with *p* < 0.05 were considered statistically significant unless otherwise specified.

## 3 Results

### 3.1 Baseline characteristics

Table 1 shows the patient demographic information and laboratory data collected within 24 h of admission. Median serum globulin was lower (24.40 vs. 26.05 g/L, *p* = 0.011) and median serum A/G was higher (1.50 vs. 1.42 g/L, *p* = 0.038) in patients with DCI than in those without, whereas serum albumin values did not differ significantly between these groups (38.45 vs. 37.80 g/L, *p* = 0.977).

**Table 1:** Baseline characteristics of patients with aSAH with and without DCI.

Variable	Total	DCI	Non-DCI	*p* value
Demographics				
Sex (%)				
Male	156 (43.0)	37 (42.5)	119 (43.1)	0.923
Female	207 (57.0)	50 (57.5)	157 (56.9)	
Age grade (%)				
1	215 (59.2)	50 (57.5)	165 (59.8)	0.702
2	148 (40.8)	37 (42.5)	111 (40.2)	
Age	57.00 (49.00, 66.50)	58.00 (48.50, 66.00)	56.00 (49.00, 67.00)	0.752
Clinical data				
Surgical grading (%)				
Grade 1	187 (51.5)	51 (58.6)	136 (49.3)	0.003*
Grade 2	116 (32.0)	32 (36.8)	84 (30.4)	
Grade 3	60 (16.5)	4 (4.6)	56 (20.3)	
Position (%)				
1	333 (93.0)	82 (94.3)	251 (92.6)	0.603
2	25 (7.0)	5 (5.7)	20 (7.4)	
Cerebral infarction (%)				
No	357 (98.3)	87 (100.0)	270 (97.8)	0.166
Yes	6 (1.7)	0 (0.0)	6 (2.2)	
Myocardial infarction (%)				
No	315 (96.9)	75 (98.7)	240 (96.4)	0.31
Yes	10 (3.1)	1 (1.3)	9 (3.6)	
GSC grading (%)				
Grade 1	81 (22.3)	17 (19.5)	64 (23.2)	0.77
Grade 2	71 (19.6)	18 (20.7)	53 (19.2)	
Grade 3	211 (58.1)	52 (59.8)	159 (57.6)	
WFNS on admission (%)				
Grade 1	146 (40.2)	34 (39.1)	112 (40.6)	0.585
Grade 2	54 (14.9)	16 (18.4)	38 (13.8)	
Grade 3	19 (5.2)	6 (6.9)	13 (4.7)	
Grade 4	97 (26.7)	23 (26.4)	74 (26.8)	
Grade 5	47 (12.9)	8 (9.2)	39 (14.1)	
Hunt–Hess on admission (%)				
Grade 1	39 (10.7)	12 (13.8)	27 (9.8)	0.679
Grade 2	134 (36.9)	27 (31.0)	107 (38.8)	
Grade 3	98 (27.0)	24 (27.6)	74 (26.8)	
Grade 4	62 (17.1)	16 (18.4)	46 (16.7)	
Grade 5	30 (8.3)	8 (9.2)	22 (8.0)	
Dilated pupils (%)				
No	338 (93.1)	80 (92.0)	258 (93.5)	0.78
Unilateral	16 (4.4)	5 (5.7)	11 (4.0)	
Bilateral	9 (2.5)	2 (2.3)	7 (2.5)	
Drinking history (%)				
No	332 (91.5)	80 (92.0)	252 (91.3)	0.85
Yes	31 (8.5)	7 (8.0)	24 (8.7)	
Smoking history (%)				
No	322 (88.7)	79 (90.8)	243 (88.0)	0.478
Yes	41 (11.3)	8 (9.2)	33 (12.0)	
Diabetes (%)				
No	330 (90.9)	81 (93.1)	249 (90.2)	0.414
Yes	33 (9.1)	6 (6.9)	27 (9.8)	
Hypertension (%)				
No	181 (49.9)	44 (50.6)	137 (49.6)	0.879
Yes	182 (50.1)	43 (49.4)	139 (50.4)	
Systolic blood pressure	156.00 (138.00, 177.00)	157.00 (136.00, 178.50)	155.00 (138.00, 175.00)	0.633
Laboratory data				
Total bilirubin	64.00 (59.85, 69.25)	63.50 (58.20, 68.50)	64.20 (60.45, 69.30)	0.298
Albumin	38.30 (34.65, 41.25)	37.80 (33.90, 42.30)	38.45 (34.73, 41.00)	0.977
Blood urea nitrogen	4.42 (3.50, 5.60)	4.56 (3.65, 5.57)	4.40 (3.48, 5.61)	0.579
Creatinine	60.30 (50.38, 73.85)	60.00 (50.80, 69.80)	60.50 (50.30, 75.20)	0.68
Blood urea nitrogen/creatinine	2.92 (1.43, 4.55)	3.10 (1.42, 4.57)	2.86 (1.44, 4.44)	0.422
Uric acid	262.50 (189.00, 342.50)	268.00 (197.50, 349.50)	261.00 (188.00, 341.00)	0.739
Glucose on admission day/day 2	8.18 (6.95, 10.00)	8.28 (7.00, 10.21)	8.12 (6.87, 9.92)	0.545
Total cholesterol	4.26 (3.62, 4.98)	4.16 (3.58, 4.87)	4.26 (3.66, 5.04)	0.257
Triglyceride	1.02 (0.72, 1.47)	1.02 (0.80, 1.51)	1.02 (0.69, 1.44)	0.446
High density lipid-cholesterol	1.20 (0.94, 1.47)	1.21 (0.88, 1.59)	1.19 (0.95, 1.45)	0.768
Low-density lipoprotein cholesterol	2.46 (1.97, 3.08)	2.37 (1.89, 2.83)	2.50 (1.98, 3.18)	0.129
Creatine kinase	150.30 (68.00, 384.00)	184.00 (72.50, 449.00)	140.10 (67.25, 333.32)	0.142
Lactate dehydrogenase	184.65 (155.00, 221.00)	204.95 (172.57, 223.65)	180.00 (150.57, 219.68)	0.006*
K	3.58 (3.29, 3.90)	3.60 (3.26, 3.91)	3.58 (3.30, 3.88)	0.924
Na	139.00 (137.00, 141.90)	139.00 (136.70, 141.40)	139.00 (137.02, 142.00)	0.345
A/G	1.44 (1.26, 1.68)	1.50 (1.31, 1.75)	1.42 (1.25, 1.65)	0.038*
Globulin	25.80 (23.10, 29.60)	24.40 (22.00, 28.00)	26.05 (23.70, 29.80)	0.011*
Phosphorus	0.82 (0.64, 1.00)	0.77 (0.61, 0.97)	0.84 (0.66, 1.02)	0.088
White blood cell	12.31 (9.70, 15.86)	12.93 (10.37, 15.25)	12.19 (9.60, 16.00)	0.342
Neutrophils	10.21 (7.56, 13.38)	10.90 (8.71, 13.43)	10.01 (7.30, 13.37)	0.135
Lymphocyte	1.30 (0.93, 1.85)	1.27 (0.84, 1.71)	1.32 (0.93, 1.88)	0.199
Red blood cell	4.42 (4.01, 4.83)	4.44 (3.98, 4.91)	4.40 (4.02, 4.80)	0.648
Hemoglobin	131.00 (117.00, 142.50)	130.00 (117.25, 144.00)	131.00 (117.00, 142.00)	0.771
Platelet	228.00 (185.00, 278.50)	226.50 (191.00, 274.25)	229.00 (184.00, 279.00)	0.739
Red blood cell volume distribution width	13.10 (12.50, 13.70)	13.20 (12.62, 13.97)	13.00 (12.50, 13.60)	0.108
Mean platelet volume	9.80 (9.10, 10.40)	9.70 (9.00, 10.28)	9.80 (9.20, 10.50)	0.148
APTT	25.70 (23.60, 28.00)	26.00 (24.40, 28.45)	25.40 (23.30, 27.65)	0.11
D-Dimer	1.52 (0.72, 3.54)	1.94 (0.66, 4.53)	1.32 (0.72, 3.28)	0.447
Fibrinogen	2.96 (2.32, 3.55)	3.00 (2.34, 3.58)	2.96 (2.32, 3.54)	0.696

Values are expressed as median (interquartile range) or number of patients (%) unless otherwise indicated. aSAH: aneurysmal subarachnoid hemorrhage; DCI, delayed cerebral ischemia; A/G, serum albumin to globulin ratio; APTT, activated partial thromboplastin time; GCS: Glasgow Coma Scale; WFNS: World Federation of Neurological Societies Scale. *p* value < 0.05 was shown in bold with the symbol “*”.

**Figure 2:**
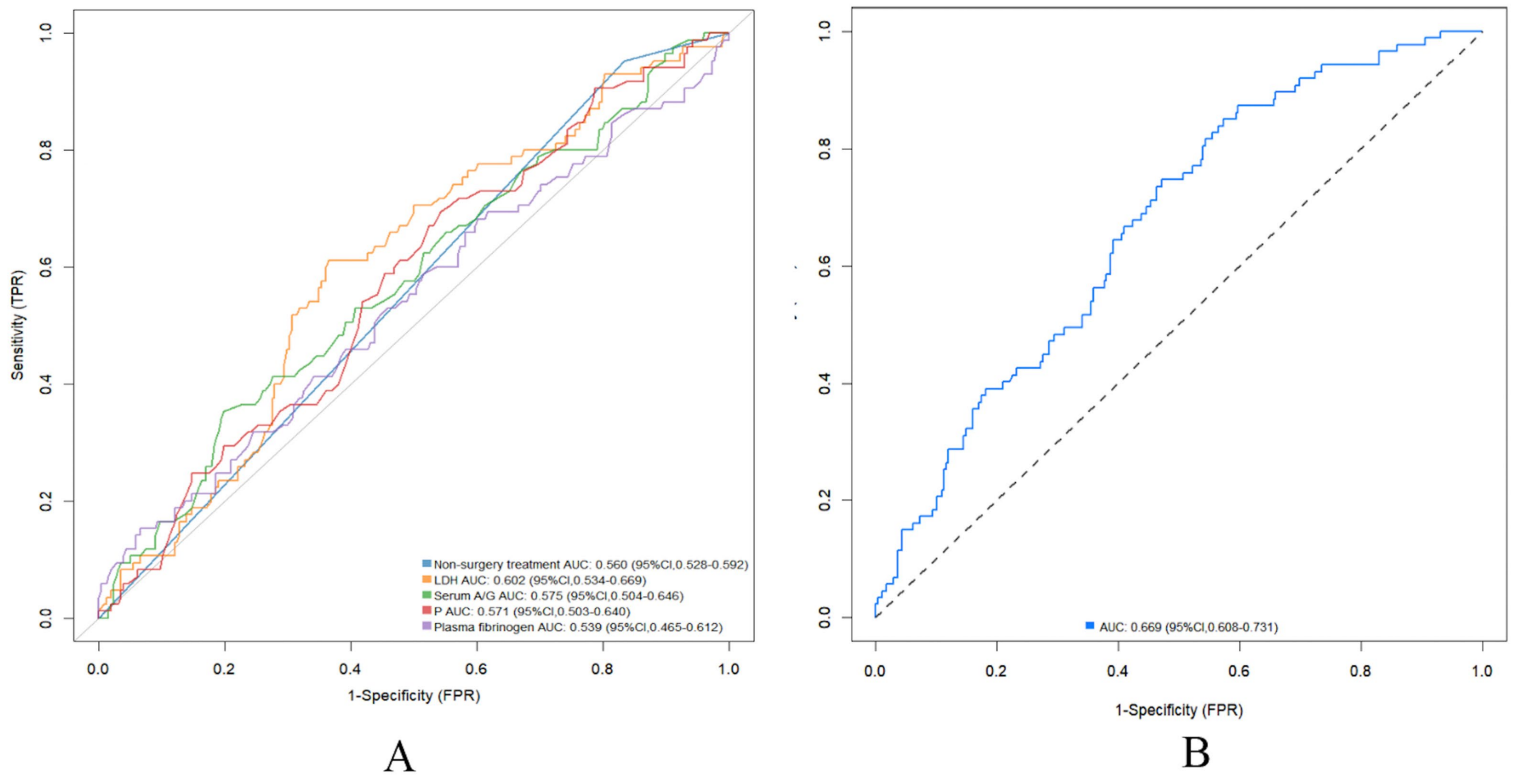
Association between serum A/G and delayed cerebral ischemia. (A) Predictive value of serum A/G, non-surgery treatment, LHD, P, and plasma fibrinogen under receiver operating characteristic curve for risk of delayed cerebral ischemia after aneurysmal subarachnoid hemorrhage separately; (B) Predictive value of serum A/G combined with non-surgery treatment, LHD, P, and plasma fibrinogen under receiver operating characteristic curve for risk of delayed cerebral ischemia after aneurysmal subarachnoid hemorrhage. A/G, serum albumin to globulin ratio; LHD, lactate dehydrogenase; P, phosphorus.

Univariate logistic regression analysis of serum A/G [odds ratio (OR), 2.336; 95% confidence interval (CI), 1.061–5.186; *p* = 0.035], non-surgical treatment (OR, 0.247; 95% CI, 0.073–0.634; *p* = 0.009), serum LDH (OR, 1.004, 95% CI, 1.000–1.007; *p* = 0.025), serum phosphorus (OR, 0.362, 95% CI, 0.140–0.902; *p* = 0.032), and plasma fibrinogen (OR, 1.251, 95% CI, 1.021–1.536; *p* = 0.03) revealed significant differences in the occurrence of DCI in patients with aSAH (Table 2). Furthermore, univariate logistic regression of DCI predictors showed that conservative treatment without surgical intervention was more likely to be administered to patients with DCI than without (OR, 0.142, 95% CI, 0.039–0.390; *p* = 0.001). Multivariable logistic regression revealed that serum A/G (OR, 2.720, 95% CI, 1.190–6.270; *p* = 0.018), non-surgical treatment (OR, 0.228, 95% CI, 0.065–0.621; *p* = 0.008), lactate dehydrogenase (OR, 1.004, 95% CI, 1.000–1.008; *p* = 0.029), phosphorous (OR, 0.354, 95% CI, 0.130–0.926; *p* = 0.038), and plasma fibrinogen (OR, 1.266, 95% CI, 1.019–1.583; *p* = 0.035) were significantly associated with DCI in patients with aSAH. The results of the ROC analysis showed that higher serum A/G, non-surgical treatment, higher lactate dehydrogenase (LDH), lower phosphorous, and higher plasma fibrinogen were associated DCI occurrence in patients with aSAH, with values of 0.575, 0.560, 0.602, 0.571, and 0.539, respectively (Figure 2A). The area under the ROC curve (AUC) for the model of combined serum A/G, nonsurgical treatment, LDH, phosphorous, and plasma fibrinogen was 0.669 (Figure 2B). Given the complexity of the multivariable logistic regression model used in the study, there is a potential risk of overfitting. We therefore adopted a 10-fold cross-validation method, wherein the data set was divided into 10 parts, to validate each prediction model and ensure generalizability. Training was performed on nine parts, while testing was conducted on the remaining one part; the process was repeated until all parts had been tested. The average AUC value of the model was 0.823, as demonstrated by the 10-fold cross-validation, proving good model diagnostic efficacy. The optimal cutoff value for serum A/G was 1.68, with a sensitivity and specificity of 67.4 and 63.5%, respectively, and a Youden index of 0.31.

**Table 2:** Multivariate logistic regression analysis of predictive factors of DCI in patients with aSAH.

	Unadjusted		Adjusted	
Variable	OR (95%CI)	*p-*value	OR (95%CI)	*p-*value
Non-Surgery treatment	0.247 (0.073–0.634)	0.009*	0.228 (0.065–0.612)	0.008*
Lactic dehydrogenase	1.004 (1.000–1.007)	0.025*	1.004 (1.000–1.008)	0.029*
Serum A/G	2.061 (0.959–4.442)	0.035*	2.720 (1.190–6.270)	0.018*
Worldwide flight services score				
Grade 1	1.061 (0.648, 1.749)	0.816	—	—
Grade 2	1.441 (0.702, 2.890)	0.309	—	—
Grade 3	1.667 (0.545, 4.650)	0.342	—	—
Grade 4	1.063 (0.568, 1.963)	0.847	—	—
Grade 5	0.889 (0.352, 2.051)	0.791	—	—
Mydriasis				
Unilateral	1.404 (0.432, 3.990)	0.54	—	—
Bilateral	1.030 (0.149, 4.575)	0.972	—	—
Systolic blood pressure	1.003 (0.995, 1.012)	0.453	—	—
Hypertension	0.977 (0.597, 1.596)	0.925	—	—
Total protein	0.979 (0.947, 1.012)	0.206	—	—
Albumin	0.995 (0.951, 1.042)	0.828	—	—
Urea nitrogen/creatinine	1.047 (0.929, 1.176)	0.448	—	—
Uric Acid	1.000 (0.998, 1.002)	0.724	—	—
Blood glucose	1.047 [0.950, 1.151]	0.346	—	—
Total cholesterol	0.850 (0.665, 1.079)	0.188	—	—
Triglyceride	0.982 (0.697, 1.349)	0.912	—	—
High-density lipoprotein	1.246 (0.661, 2.329)	0.493	—	—
Low density lipoprotein	0.817 (0.613, 1.075)	0.158	—	—
Creatine kinase	1.000 (1.000, 1.001)	0.097	—	—
K	0.851 (0.511, 1.399)	0.528	—	—
Na	0.956 (0.902, 1.010)	0.112	—	—
P	0.362 (0.140, 0.902)	0.032*	0.354 (0.130, 0.926)	0.038*
White blood cell	1.006 (0.966, 1.044)	0.755	—	—
Neutrophil	1.032 (0.982, 1.085)	0.21	—	—
Lymphocyte	0.853 (0.645, 1.083)	0.225	—	—
Monocyte	1.078 (0.568, 1.977)	0.812	—	—
Erythrometry	1.256 (0.840, 1.891)	0.27	—	—
Hemoglobin	1.005 (0.992, 1.018)	0.47	—	—
Platelet	0.705 (0.230, 1.797)	0.497	—	—
Red blood cell distribution width	1.029 (1.002, 1.151)	0.584	—	—
Mean platelet volume	0.864 (0.680, 1.085)	0.219	—	—
APTT	1.001 (0.948, 1.054)	0.965	—	—
D-dimer	1.026 (0.984, 1.068)	0.206	—	—
Plasma fibrinogen	1.251 (1.021, 1.536)	0.03*	1.266 (1.019, 1.583)	0.035*

DCI, delayed cerebral ischemia; OR, odds ration; LDH, lactate dehydrogenase; Na, sodium; A/G, serum albumin to globulin ratio; P, phosphorus; APTT, Activated partial thromboplastin time. *p* value < 0.05 was shown in bold with the symbol “*”.

## 4 Discussion

**Figure 3:**
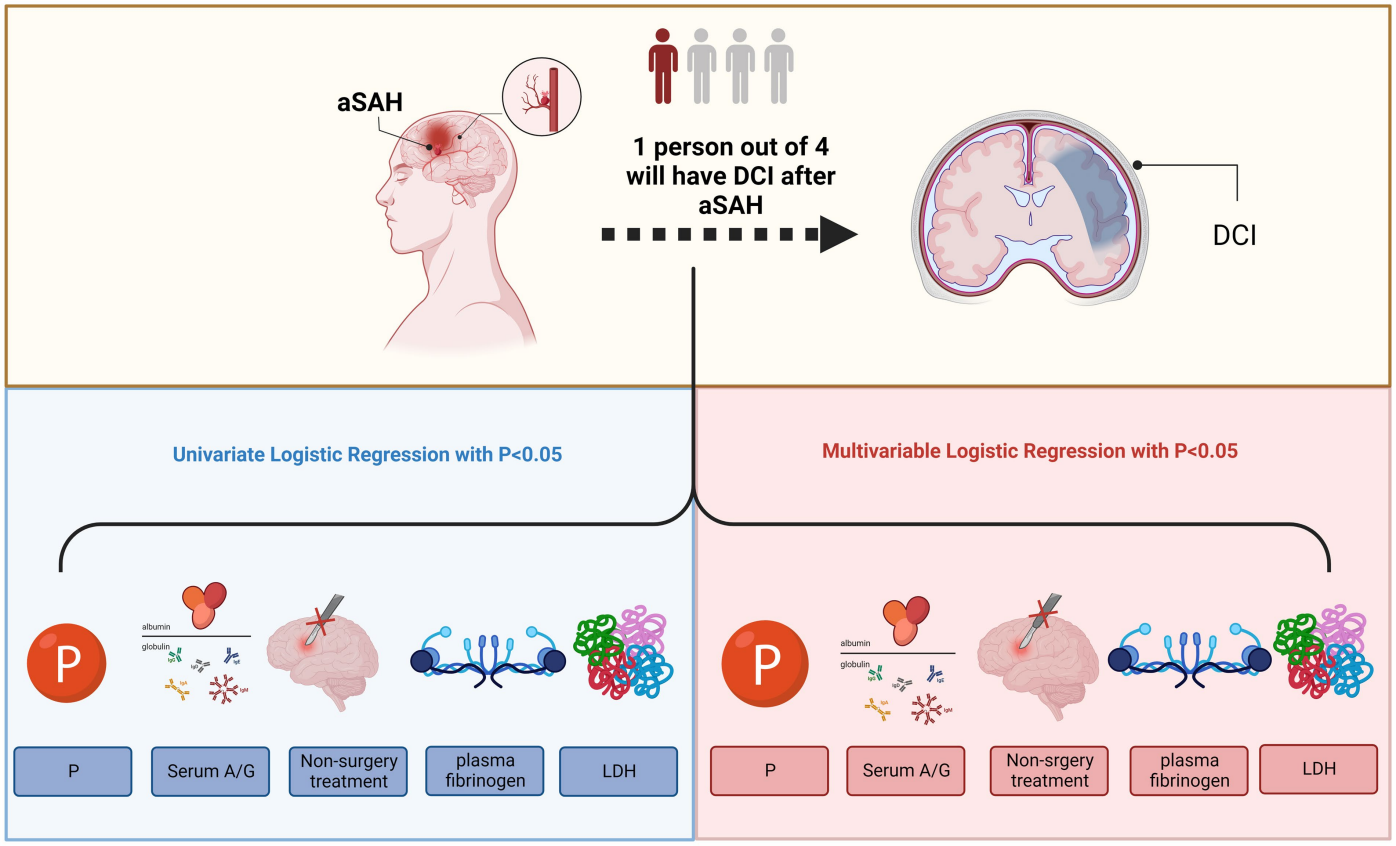
Higher serum A/G, non-surgery treatment, higher LHD, lower P, and higher plasma fibrinogen were associated with the occurrence of delayed cerebral ischemia after aneurysmal subarachnoid hemorrhage. A/G, serum albumin to globulin ratio; LHD, lactate dehydrogenase; P, phosphorus. This image was created with BioRender.com.

In this study, we aimed to determine whether blood A/G levels were associated with the development of DCI in patients with aSAH, and found a significant relationship between the prevalence of DCI and higher serum A/G ratios in patients with aSAH (Figure 3). The correlation strength was further improved when LDH, phosphorous, plasma fibrinogen, and non-surgical treatment were included in the multivariable logistic regression analysis, increasing the correlation with DCI.

SAH can progress suddenly and rapidly, especially in patients with severe illness upon admission and those who experience prolonged hospital stays (15). Therefore, predicting the prognosis of aSAH remains a clinical challenge (4). Our findings suggest that a high serum A/G ratio (> 1.68) is associated with DCI after aSAH. The pathogenesis of DCI includes cerebral vasospasm, early brain injury, microembolus formation, cortical diffusion, and depolarization (16). Cerebral arteries are frequently narrowed in patients with aSAH and can be detected by transcranial Doppler ultrasonography (TCD) (17), CTA (18), or electroencephalography (19). While DSA and TCD can diagnose vasospasms when they occur, there are currently no specific objective methods to predict the development of DCI (20). Therefore, we investigated serum A/G, which indicates nutritional and inflammatory states, as a novel, readily available, and noninvasive indicator that associate with DCI after aSAH.

The study’s finding that a high A/G ratio is associated with a higher incidence of DCI contradicts established literature, where low A/G ratios are typically linked with poor outcomes in various conditions, such as ischemic stroke (12) and cancer (21). This may be due to the disrupted mitochondrial respiration and Fenton reaction activated by blood debris, which increases the generation of reactive oxygen species (ROS) (22). Proteins that are abundant in biological systems are major targets for ROS and most susceptible to oxidative damage (23). Therefore, oxidized albumin levels increase after subarachnoid hemorrhage, which may induce endothelial injury and contribute to delayed cerebral vasospasm and DCI (23, 24). Additionally, high concentrations of albumin were associated with increased levels of glutathione disulfide and elevated ROS production in stimulated endothelial cells, which may play a pro-oxidative role in the pathophysiology of aSAH (25). Furthermore, high concentrations of albumin decreased the expression of Heme oxygenase-1 (HO-1), an enzyme that removes potentially toxic molecules. HO-1 serves as a lipid-soluble delivery form of iron and may have pro-oxidative capacity by controlling the redox state of cys-34-thiols (19). These factors contribute to endothelial dysfunction, thereby triggering DCI (26). Serum globulins, as a mixture of proteins, include immunoglobulins and complement, which play a defensive role and reflect the collective immune status (27). Lower levels of globulin may indicate reduced body’s defenses, which lead to poor outcomes (28).

Multivariate analysis showed that non-surgical treatment, LDH, phosphorous, and plasma fibrinogen were significant indicators of DCI after aSAH. In this study, higher serum LDH levels were associated with DCI, which is consistent with a previous report (29). LDH is a key enzyme in anaerobic glycolysis that catalyzes the production of lactate from pyruvate (30). SAH is usually accompanied by hypoxia, and higher LDH levels may contribute to the rapid accumulation of lactate, which may compromise vascular endothelium and lead to DCI (31). Additionally, we found that non-surgical treatment was associated with the occurrence of DCI after aSAH. This may be explained by surgical removal of hematoma, which can result in vasospasm and subsequent DCI (32). Vascular endothelial damage and microthrombosis are important factors in DCI development after aSAH (24). However, fibrinogen is a coagulation factor that accelerates the process of thrombosis, which can ultimately lead to DCI (33, 34). Studies have reported that the immune function of patients with hypophosphatemia is significantly lower than that of healthy individuals; additionally, hypophosphatemia is associated with poor outcome and might have the potential to lead to a poor outcome in SAH (35).

This study had some limitations. First, this was a retrospective study; therefore, any conclusions are subject to study design limitations, including recall and observer bias. To address these issues, future multicenter prospective studies are warranted. Second, due to the relatively small sample size in this study, no significant relationship was detected between DCI and WFNS. Future studies with larger sample sizes are warranted for more reliable results. Third, the volume of the admission subarachnoid clot was not evaluated in this study, which is strongly associated with DCI. Further studies are needed to address this issue and provide more reliable results. Fourth, serum A/G values were not consistently monitored during follow-up. Therefore, we could not determine whether changes in these values could affect the likelihood of DCI occurrence after aSAH. Consequently, further investigation is required on this issue. Finally, the area under the ROC curve of the serum A/G in this study was relatively small, which may have compromised the power of our primary results.

## 5 Conclusion

Serum A/G values are associated with DCI among patients with aSAH. Elevated serum A/G values upon admission might be associated with DCI occurrence, which could facilitate the early identification of patients with aSAH at high risk for DCI. Further studies with larger sample sizes are required to confirm our findings.

## Data Availability

The original contributions presented in the study are included in the article. Further inquiries can be directed to the corresponding author/s.

## References

[1] van Gijn J Kerr RS Rinkel GJ. Subarachnoid haemorrhage. Lancet. (2007) 369:306–18. doi: 10.1016/S0140-6736(07)60153-6 17258671

[2] Etminan N Chang HS Hackenberg K de Rooij NK Vergouwen MDI Rinkel GJE. Worldwide incidence of aneurysmal subarachnoid hemorrhage according to region, time period, blood pressure, and smoking prevalence in the population: a systematic review and Meta-analysis. JAMA Neurol. (2019) 76:588–97. doi: 10.1001/jamaneurol.2019.0006, PMID: 30659573 PMC6515606

[3] Vergouwen MD Vermeulen M van Gijn J Rinkel GJ Wijdicks EF Muizelaar JP. Definition of delayed cerebral ischemia after aneurysmal subarachnoid hemorrhage as an outcome event in clinical trials and observational studies: proposal of a multidisciplinary research group. Stroke. (2010) 41:2391–5. doi: 10.1161/STROKEAHA.110.589275, PMID: 20798370

[4] Hoh BL Ko NU Amin-Hanjani S Chou S-Y Cruz-Flores S Dangayach NS. 2023 guideline for the Management of Patients with Aneurysmal Subarachnoid Hemorrhage: a guideline from the American Heart Association/American Stroke Association. Stroke. (2023) 54:e314–70. doi: 10.1161/STR.0000000000000436, PMID: 37212182

[5] De Simone G di Masi A Ascenzi P. Serum albumin: a multifaced enzyme. Int J Mol Sci. (2021) 22:10086. doi: 10.3390/ijms221810086, PMID: 34576249 PMC8466385

[6] Phillips A Shaper AG Whincup PH. Association between serum albumin and mortality from cardiovascular disease, cancer, and other causes. Lancet. (1989) 2:1434–6. doi: 10.1016/S0140-6736(89)92042-4, PMID: 2574367

[7] Ohashi SN DeLong JH Kozberg MG Mazur-Hart DJ van Veluw SJ Alkayed NJ. Role of inflammatory processes in hemorrhagic stroke. Stroke. (2023) 54:605–19. doi: 10.1161/STROKEAHA.122.037155, PMID: 36601948

[8] Xie HL Zhang Q Ruan GT Ge YZ Hu CL Song MM. Evaluation and validation of the prognostic value of serum albumin to globulin ratio in patients with Cancer Cachexia: results from a large multicenter collaboration. Front Oncol. (2021) 11:707705. doi: 10.3389/fonc.2021.707705, PMID: 34568033 PMC8461248

[9] Zhang J Wang T Fang Y Wang M Liu W Zhao J. Clinical significance of serum albumin/globulin ratio in patients with pyogenic liver abscess. Front Surg. (2021) 8:677799. doi: 10.3389/fsurg.2021.677799, PMID: 34917645 PMC8669143

[10] Rahman MZ Begum BA. Serum total protein, albumin and a/G ratio in different grades of protein energy malnutrition. Mymensingh Med J. (2005) 14:38–40. PMID: 15695952

[11] Maeda S Takeya Y Oguro R Akasaka H Ryuno H Kabayama M. Serum albumin/globulin ratio is associated with cognitive function in community-dwelling older people: the septuagenarians, octogenarians, nonagenarians investigation with centenarians study. Geriatr Gerontol Int. (2019) 19:967–71. doi: 10.1111/ggi.13751, PMID: 31461209

[12] Wang A Zhang Y Xia G Tian X Zuo Y Chen P. Association of serum albumin to globulin ratio with outcomes in acute ischemic stroke. CNS Neurosci Ther. (2023) 29:1357–67. doi: 10.1111/cns.14108, PMID: 36794538 PMC10068453

[13] Skrivankova VW Richmond RC Woolf BAR Yarmolinsky J Davies NM Swanson SA. Strengthening the reporting of observational studies in epidemiology using Mendelian randomization: the STROBE-MR statement. JAMA. (2021) 326:1614–21. doi: 10.1001/jama.2021.18236, PMID: 34698778

[14] Connolly ES Jr Rabinstein AA Carhuapoma JR Derdeyn CP Dion J Higashida RT. Guidelines for the management of aneurysmal subarachnoid hemorrhage: a guideline for healthcare professionals from the American Heart Association/american Stroke Association. Stroke. (2012) 43:1711–37. doi: 10.1161/STR.0b013e3182587839 22556195

[15] Lauzier DC Jayaraman K Yuan JY Diwan D Vellimana AK Osbun JW. Early brain injury after subarachnoid hemorrhage: incidence and mechanisms. Stroke. (2023) 54:1426–40. doi: 10.1161/STROKEAHA.122.040072 36866673 PMC10243167

[16] Geraghty JR Testai FD. Delayed cerebral ischemia after subarachnoid hemorrhage: beyond vasospasm and towards a multifactorial pathophysiology. Curr Atheroscler Rep. (2017) 19:50. doi: 10.1007/s11883-017-0690-x 29063300

[17] Westermaier T Pham M Stetter C Willner N Solymosi L Ernestus RI. Value of transcranial Doppler, perfusion-CT and neurological evaluation to forecast secondary ischemia after aneurysmal SAH. Neurocrit Care. (2014) 20:406–12. doi: 10.1007/s12028-013-9896-0, PMID: 23982597

[18] Smith NM Sweeney EM Gupta A Patsalides A Sanelli P Ivanidze J. Diagnostic accuracy of shuttle CT angiography (CTA) and helical CTA in the diagnosis of vasospasm. Clin Imaging. (2022) 81:37–42. doi: 10.1016/j.clinimag.2021.09.004, PMID: 34598002

[19] Scherschinski L Catapano JS Karahalios K Koester SW Benner D Winkler EA. Electroencephalography for detection of vasospasm and delayed cerebral ischemia in aneurysmal subarachnoid hemorrhage: a retrospective analysis and systematic review. Neurosurg Focus. (2022) 52:E3. doi: 10.3171/2021.12.FOCUS21656, PMID: 35231893

[20] Geraghty JR Lung TJ Hirsch Y Katz EA Cheng T Saini NS. Systemic immune-inflammation index predicts delayed cerebral vasospasm after aneurysmal subarachnoid hemorrhage. Neurosurgery. (2021) 89:1071–9. doi: 10.1093/neuros/nyab354, PMID: 34560777 PMC8600162

[21] Xia Z Fu X Yuan X Li J Wang H Sun J. Serum albumin to globulin ratio prior to treatment as a potential non-invasive prognostic indicator for urological cancers. Front Nutr. (2022) 9:1012181. doi: 10.3389/fnut.2022.1012181, PMID: 36386921 PMC9643875

[22] Yang Y Chen S Zhang JM. The updated role of oxidative stress in subarachnoid hemorrhage. Curr Drug Deliv. (2017) 14:832–42. doi: 10.2174/1567201813666161025115531, PMID: 27784210

[23] Luna C Alique M Navalmoral E Noci MV Bohorquez-Magro L Carracedo J. Aging-associated oxidized albumin promotes cellular senescence and endothelial damage. Clin Interv Aging. (2016) 11:225–36. doi: 10.2147/CIA.S91453, PMID: 27042026 PMC4780186

[24] Dodd WS Laurent D Dumont AS Hasan DM Jabbour PM Starke RM. Pathophysiology of delayed cerebral ischemia after subarachnoid hemorrhage: a review. J Am Heart Assoc. (2021) 10:e021845. doi: 10.1161/JAHA.121.021845, PMID: 34325514 PMC8475656

[25] Imber M Pietrzyk-Brzezinska AJ Antelmann H. Redox regulation by reversible protein S-thiolation in gram-positive bacteria. Redox Biol. (2019) 20:130–45. doi: 10.1016/j.redox.2018.08.017, PMID: 30308476 PMC6178380

[26] Kremer H Baron-Menguy C Tesse A Gallois Y Mercat A Henrion D. Human serum albumin improves endothelial dysfunction and survival during experimental endotoxemia: concentration-dependent properties. Crit Care Med. (2011) 39:1414–22. doi: 10.1097/CCM.0b013e318211ff6e, PMID: 21336119

[27] Revello MG Lazzarotto T Guerra B Spinillo A Ferrazzi E Kustermann A. A randomized trial of hyperimmune globulin to prevent congenital cytomegalovirus. N Engl J Med. (2014) 370:1316–26. doi: 10.1056/NEJMoa1310214, PMID: 24693891

[28] Suratannon N Tantithummawong P Hurst CP Chongpison Y Wongpiyabovorn J van Hagen PM. Pediatric prediction model for low immunoglobulin G level based on serum globulin and illness status. Front Immunol. (2022) 13:825867. doi: 10.3389/fimmu.2022.825867 35265080 PMC8899039

[29] Zan X Deng H Zhang Y Wang P Chong W Hai Y. Lactate dehydrogenase predicting mortality in patients with aneurysmal subarachnoid hemorrhage. Ann Clin Transl Neurol. (2022) 9:1565–73. doi: 10.1002/acn3.51650, PMID: 35984334 PMC9539376

[30] Certo M Tsai CH Pucino V Ho PC Mauro C. Lactate modulation of immune responses in inflammatory versus tumour microenvironments. Nat Rev Immunol. (2021) 21:151–61. doi: 10.1038/s41577-020-0406-2, PMID: 32839570

[31] Cobben NA Drent M Schols AM Lamers RJ Wouters EF Van Dieijen-Visser MP. Serum lactate dehydrogenase and its isoenzyme pattern in ex-coalminers. Respir Med. (1997) 91:616–23. doi: 10.1016/S0954-6111(97)90008-1, PMID: 9488895

[32] Lu J Wang L Li R Lin F Chen Y Yan D. Timing of operation for poor-grade aneurysmal subarachnoid hemorrhage: relationship with delayed cerebral ischemia and poor prognosis. CNS Neurosci Ther. (2023) 29:1120–8. doi: 10.1111/cns.14088, PMID: 36627811 PMC10018093

[33] Maresca G Di Blasio A Marchioli R Di Minno G. Measuring plasma fibrinogen to predict stroke and myocardial infarction: an update. Arterioscler Thromb Vasc Biol. (1999) 19:1368–77. doi: 10.1161/01.ATV.19.6.1368 10364066

[34] Sato S Iso H Noda H Kitamura A Imano H Kiyama M. Plasma fibrinogen concentrations and risk of stroke and its subtypes among Japanese men and women. Stroke. (2006) 37:2488–92. doi: 10.1161/01.STR.0000242473.13884.8e, PMID: 16946147

[35] Haider DG Lindner G Wolzt M Ahmad SS Sauter T Leichtle AB. Hyperphosphatemia is an independent risk factor for mortality in critically ill patients: results from a cross-sectional study. PLoS One. (2015) 10:e0133426. doi: 10.1371/journal.pone.0133426, PMID: 26252874 PMC4529074

